# Supplementary material for: Previous motor task performance impacts phase-based EEG resting-state connectivity states
Source: Imaging Neurosci (Camb). 2024 Mar 14;2:imag-2-00109. doi: 10.1162/imag_a_00109 (PMC12247568; doi:10.1162/imag_a_00109)
Supplement: Supplementary Material [file imag_a_00109-supp.pdf]

Supplementary Material

Previous motor task performance impacts phase-based EEG resting-state connectivity states

Nils Rosjat[1], Maximilian Hommelsen[1], Gereon R. Fink[1,2], Silvia Daun[1,3,*]

1 Cognitive Neuroscience, Institute of Neuroscience and Medicine (INM-3), Forschungszentrum Jülich, Jülich, Germany
2 Medical Faculty, University of Cologne, and Department of Neurology, University Hospital Cologne, Cologne, Germany
3 Institute of Zoology, University of Cologne, Cologne, Germany

S1 - Choice of sliding window size In optimizing our sliding window approach, selecting an appropriate window length played a crucial role in capturing the dynamic nature of the neural connectivity networks. To determine the optimal window length, a series of calculations were performed for varying window sizes, ranging from 50 ms to 600 ms, as depicted in Fig. S1. In the pursuit of aligning the window size with reported microstate coverages and leveraging the high temporal resolution of EEG data, a shorter window size initially seemed desirable. However, a notable trend emerged upon closer examination of the results obtained with the shorter window lengths. In particular, it was observed that within the alpha frequency range, almost all potential connections exhibited values approaching the maximum limit of 1. The pronounced trend of connectivity values approaching the maximum limit of 1 at shorter window lengths is a direct consequence of reduced variance in phase differences. This phenomenon, inherent to the use of shorter sliding window lengths, leads to a systematic inflation of the phase synchronization connectivity metric, which is a pivotal measure in our analysis. Consequently, this phenomenon led to a limited variation in the derived networks, thereby hindering the ability to discern meaningful connectivity patterns. Acknowledging this methodological artifact, our strategy to extend window lengths aimed to introduce greater variance in phase differences. Such variance is critical for differentiating genuine connectivity patterns from spurious synchronizations that might arise due to the temporal granularity of the analysis.

Given these observations, a judicious compromise was necessary to balance variance and connectivity strength within the derived networks. After careful analysis, a window length of 300 ms emerged as an optimal choice. The decision to use a 300 ms window was empirically driven and characterized by an inflection point in our analyses where the mean connectivity strength began to show a meaningful decrease. This point represents a balance, capturing a broad range of connectivity strengths — from as low as 0.2 to the upper limit of 1.0 — thereby offering a nuanced view of network dynamics. At this 300 ms point, we observed not only a decrease in mean connectivity strength, indicative of mitigating the earlier noted saturation effect, but also an increase in the variability of connectivity measurements. This variability is essential for discerning the complex, transient nature of neural interactions, especially within

the highly dynamic alpha frequency range. By adopting this window length, we effectively captured dynamic variations in connectivity while maintaining a level of variance conducive to meaningful network analysis. Furthermore, the choice of a 300 ms window preserves the temporal resolution crucial for EEG data analysis. It ensures that while we extend the window to reduce metric inflation, we do not compromise on capturing the fast-evolving nature of neural networks, thus maintaining a delicate balance between temporal specificity and analytical robustness.

S2 - Microstate topographies remain stable over measurement sessions In the main text, we presented the four MS topographies as defined by all four sessions combined. To check whether these states are stable across all measurement sessions, i.e., days and between RS1 and RS2, we performed the MS clustering separately for each measurement day. When comparing the MS topographies in the main text (Fig. S2, first row, RS1) with the other four MS topography configurations (second to fifth row, session 1 - 4, RS1) and RS2 (sixth row), there is a high level of similarity with only minor changes in the MS topographies. Therefore, we can assume that the MS topographies do not change significantly over the measurement days.

S3 - Microstate coverages in alpha frequency band Here, we present additional analyses of microstate coverages within the alpha frequency band (8-12 Hz) for microstates A, B, C, and D (Fig. S3). This analysis complements our results presented in the main text, which focused on broadband microstate analysis. By filtering the signal to isolate the alpha frequency band, we aimed to discern any differences for specific microstates (MS) during the individual sessions as well as for resting states RS1 and RS2. Our findings indicate a consistent pattern with the broadband analysis; that is, there is no evident preference for any microstate dominating the coverage in either of the sessions (A) or resting states (B). This uniform distribution of microstate coverage across MS A, MS B, MS C, and MS D in the alpha band mirrors the results observed in the broadband analysis (cf. Fig. 3D and E), underscoring our decision to exclude these specific findings from the main manuscript.

S4 - Source reconstruction - motor task To ensure the validity of the source localization in the resting-states, we first performed a localization of the motor activity during finger movement in the "Tap" task. As shown in Fig. S4, there is an increase in activity in the area of the primary motor cortex during finger movements. Furthermore, we used this experimental condition to determine the best possible parcellation for our data. The aim was to map the activity to as fine structures as possible, which are close to the course of the source activity, but not to determine too many sources simultaneously since only 61 underlying EEG recordings were available. While the Desikan-Killany atlas and the Brodmann parcellation are coarse and not close to the observed activity, the sub-parcellation of the DKT atlas clearly defines too many sources. The Destrieux atlas is close to the observed activity and has hardly more parcellations than the Brodman atlas. Thus, we selected the Destrieux parcellation for our further investigations.

S5 - Source connectivity - resting-state Fig. S5 provides a comprehensive exemplary view of the mean corrected imaginary phase-locking value (ciPLV) across all subjects, sessions, and time windows for each pair of source parcels under investigation. The purpose of this figure is to demonstrate the existence of varying connectivity strengths throughout the entire network, indicating that the connections between these parcels are not zero. This observation is crucial as it signifies the presence of meaningful connectivity within the network. By illustrating the non-zero connectivity, this figure supports the notion that the networks formed based on these connections carry significance and are not merely random or spurious associations.

S6 - Connectivity state analysis for 8 states Besides the analysis defining 4 connectivity states, we clustered the data into 8 connectivity states (CS). The results are presented in Fig. S6. We generally observed similar phenomena as in the results presented in the main text. We found 5 states (CS A, B, C, G, and H) that occurred roughly 10% of the time, while three other states showed an increased duration of 15% (CS E), 20% (CS F), and 28% (CS D). The same behavior could be observed when dividing the data into measurement sessions. When examining the patterns of the CS, it is noticeable that there are remarkable similarities to the phenomena described in the main text (e.g., CS C, E, and G). Besides the patterns already described, 4 other patterns appeared, which in some cases (such as CS A and CS C) were in part very similar to each other, and thus, one should be careful in arguing that they represent new/independent states.

S7 - Connectivity state metrics Additionally to our analysis of CS coverages, we explored CS frequency, contribution and duration. We observed that frequency and contribution share characteristics akin to CS duration. For this reason, we show the results on the connectivity state duration only, which is defined as the average duration for which the network persistently remains in a single connectivity state, see Fig. S7. Our observations revealed consistent patterns across all four measurement days (Fig. S7 A and B). For both RS1 and RS2, this metric was comparable to the CS coverage. Notably, in RS1, the CS D remained the dominant state in duration, mirroring its prevalence in total coverage (Fig. S7 A and C). In RS2, though the coverage indicated considerable variance for CS C, its average duration was extended, whereas the duration for CS D was diminished (Fig. S7 C). The overall consistency of this metric reaffirms the reproducibility of our methodological approach.

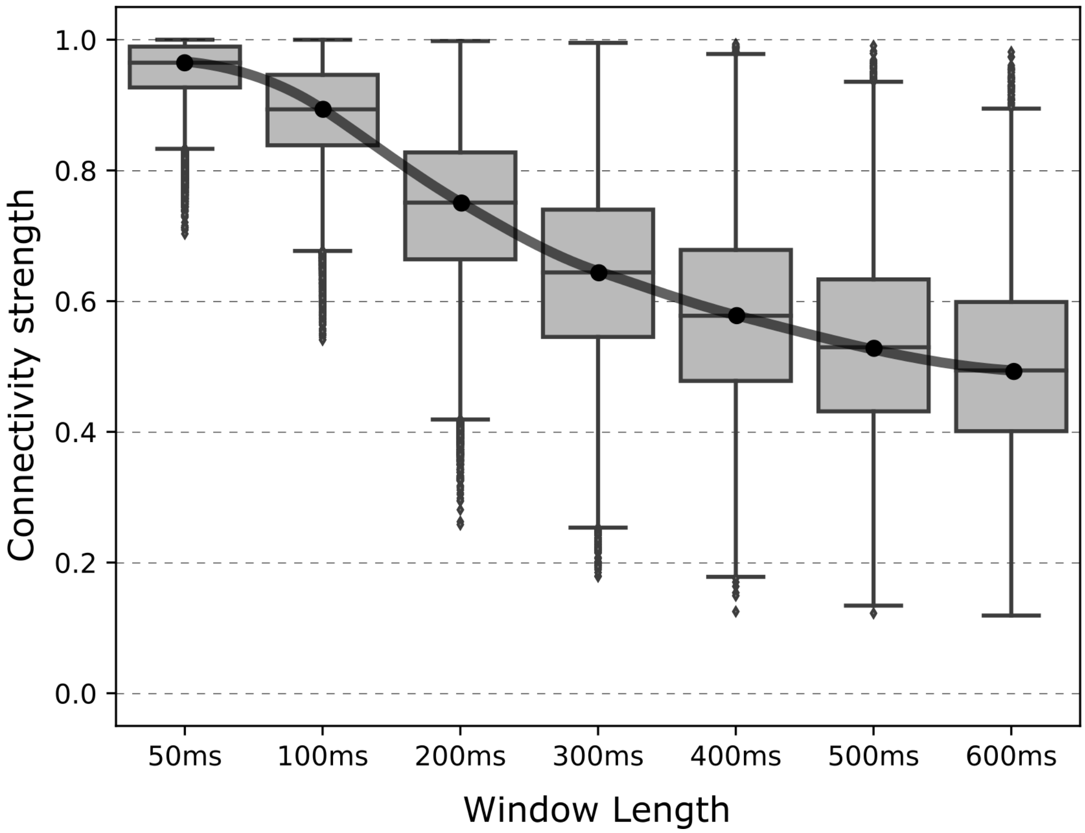

Fig S1. Connectivity strength distribution for various window lengths. Distribution of all connectivity values for various sliding window lengths ranging from 50 ms (left) to 600 ms (right).

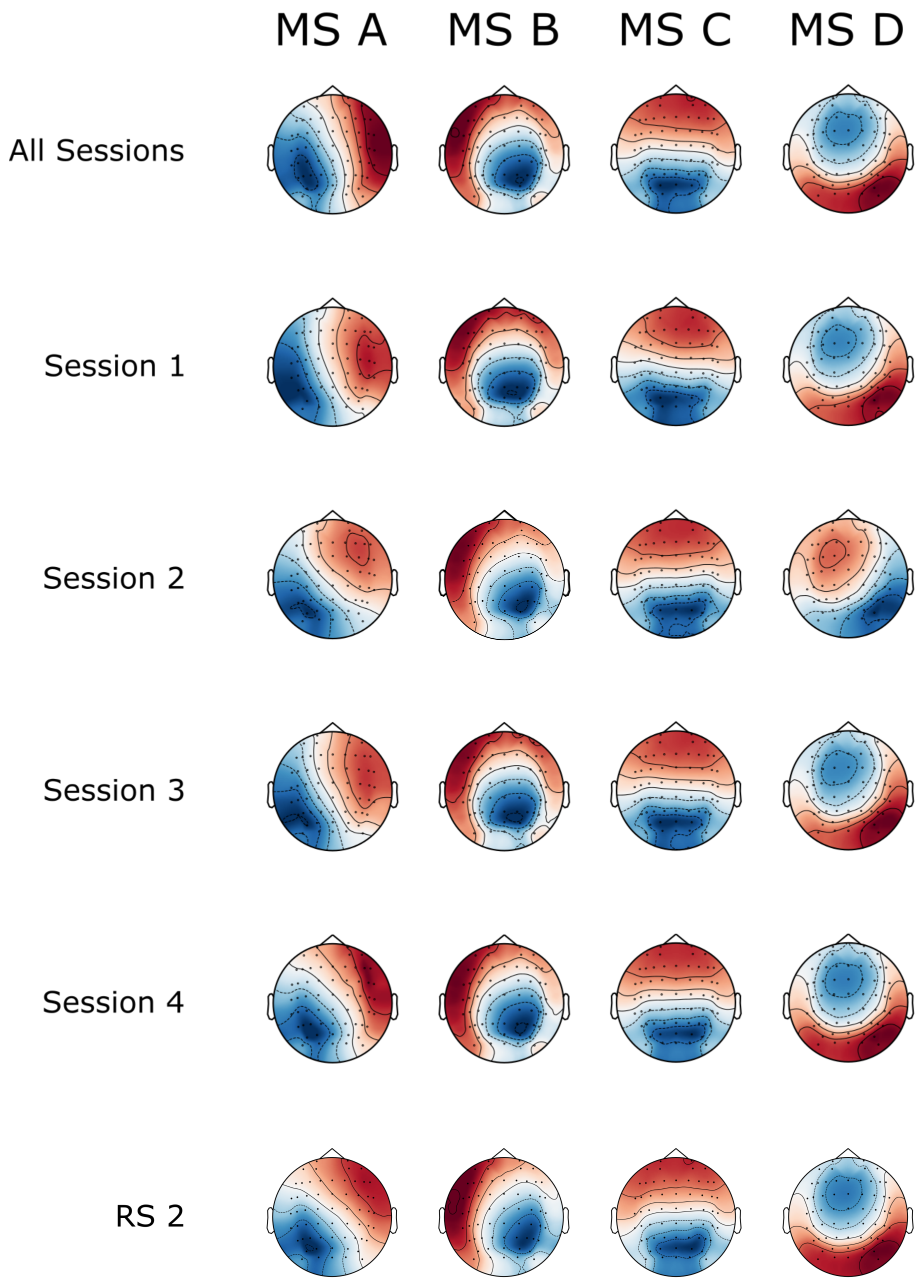

Fig S2. MS topographies remain stable over measurement sessions. Microstate topographies analyzed separately for different measurement session, i.e. days. First row: MS topography for all sessions as shown in the main text (RS1); Second row: MS topography for session 1, RS1; Third row: MS topography for session 2, RS1; Fourth row: MS topography for session 3, RS1; Fifth row: MS topography for session 4, RS1; Sixth row: MS topography for RS2, all sessions.

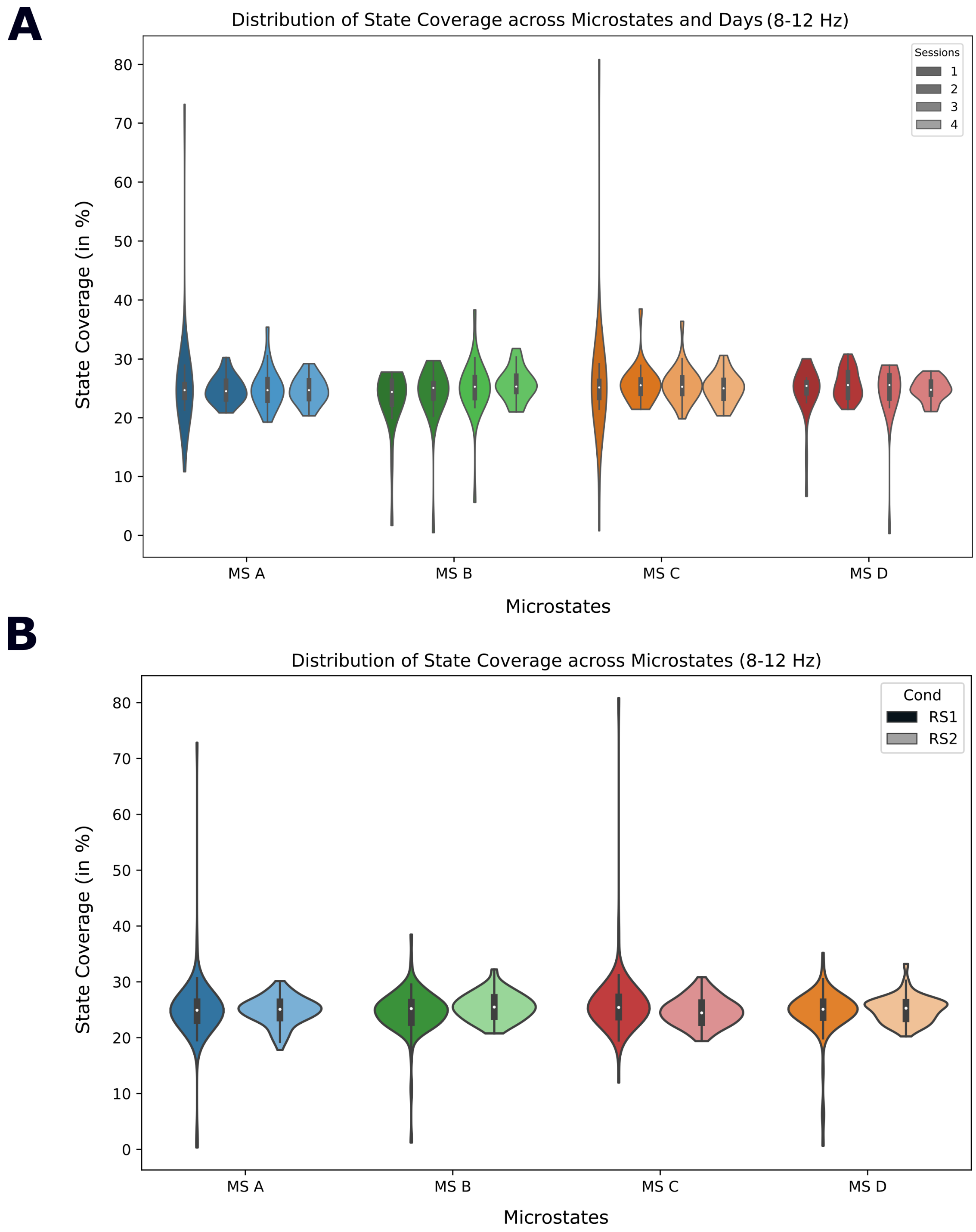

Fig S3. MS coverage distribution remains stable for alpha frequencies. Alpha frequency MS coverages for states A-D in A) session 1-4 and B) in RS1 and RS2.

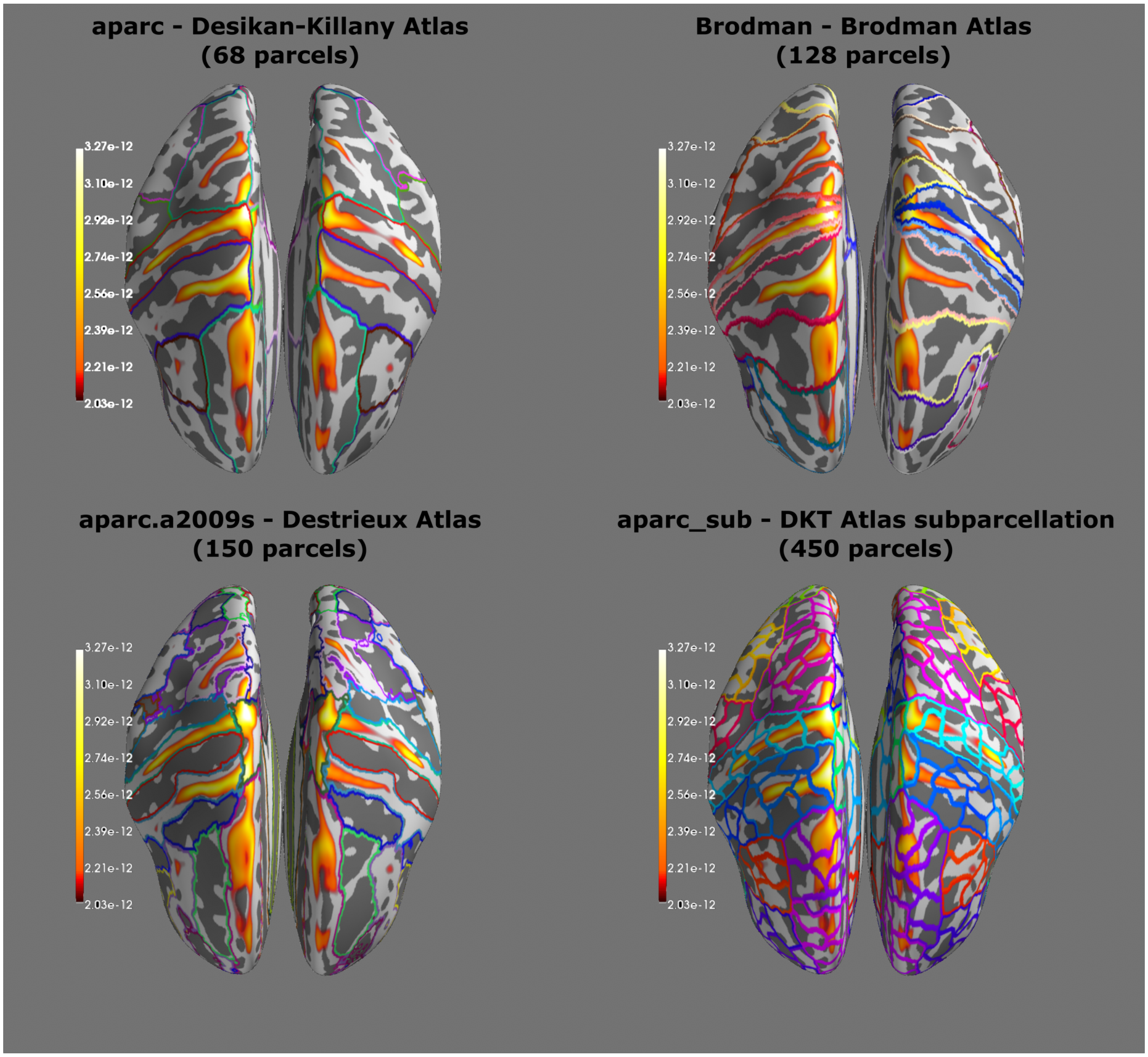

Fig S4. Source reconstructed activity for finger tap movement. Source reconstructed motor activity for four different types of cortical parcellations. Top left: Desikan-Killany atlas; Top right: Brodmann atlas; Bottom left: Destrieux atlas; Bottom right: DKT atlas subparcellation.

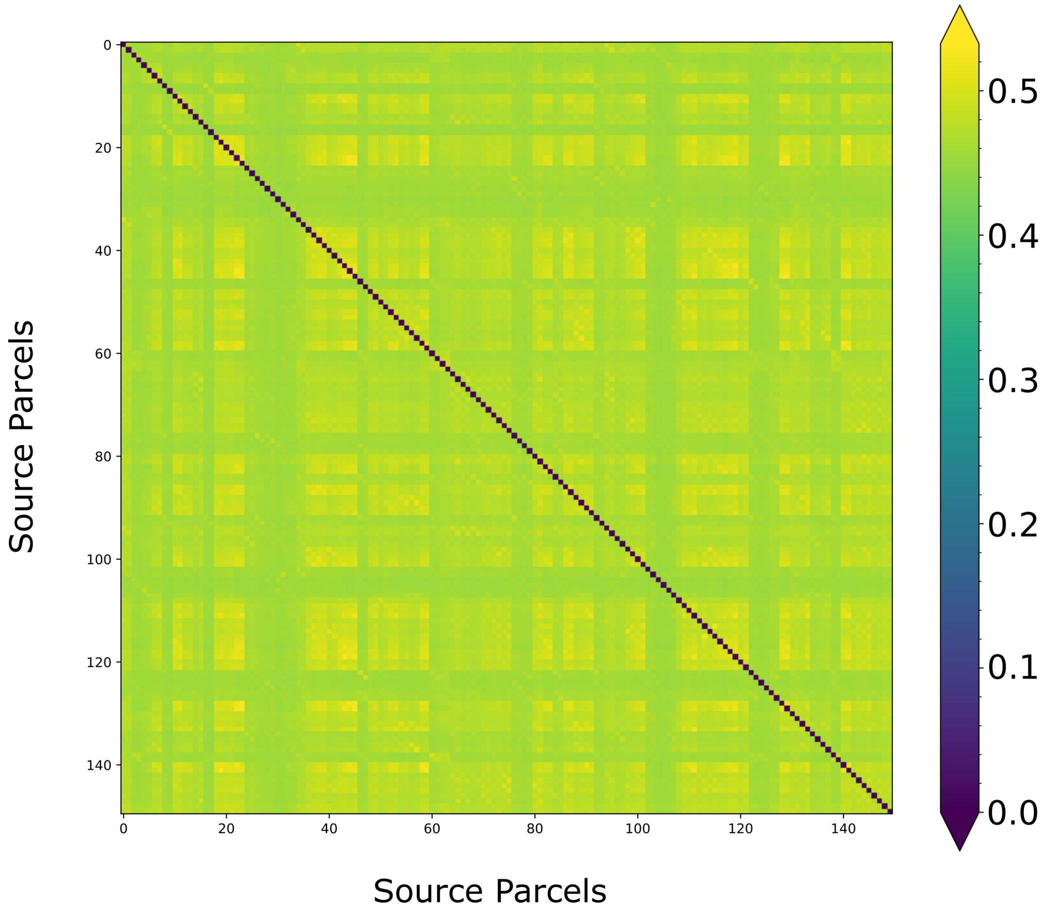

Fig S5. Source ciPLV. Mean ciPLV over all subjects, sessions, and time windows of RS1 and RS2 for all pairs of source parcels in consideration.

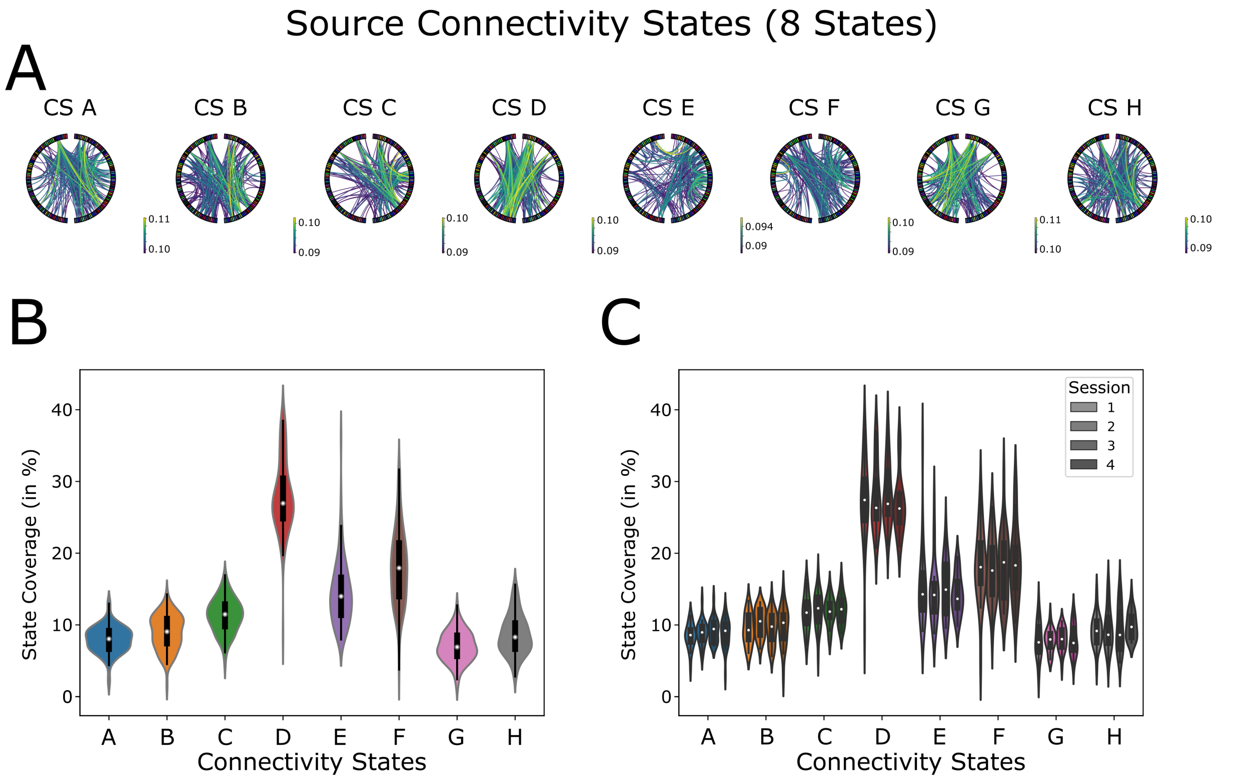

Fig S6. CS analysis for 8 states. Source connectivity state analysis (A-C) for 8 states in RS1. A: CS networks for CS A-H; B: CS coverages for CS A-H; C: CS coverages for CS A-H for different measurement sessions.

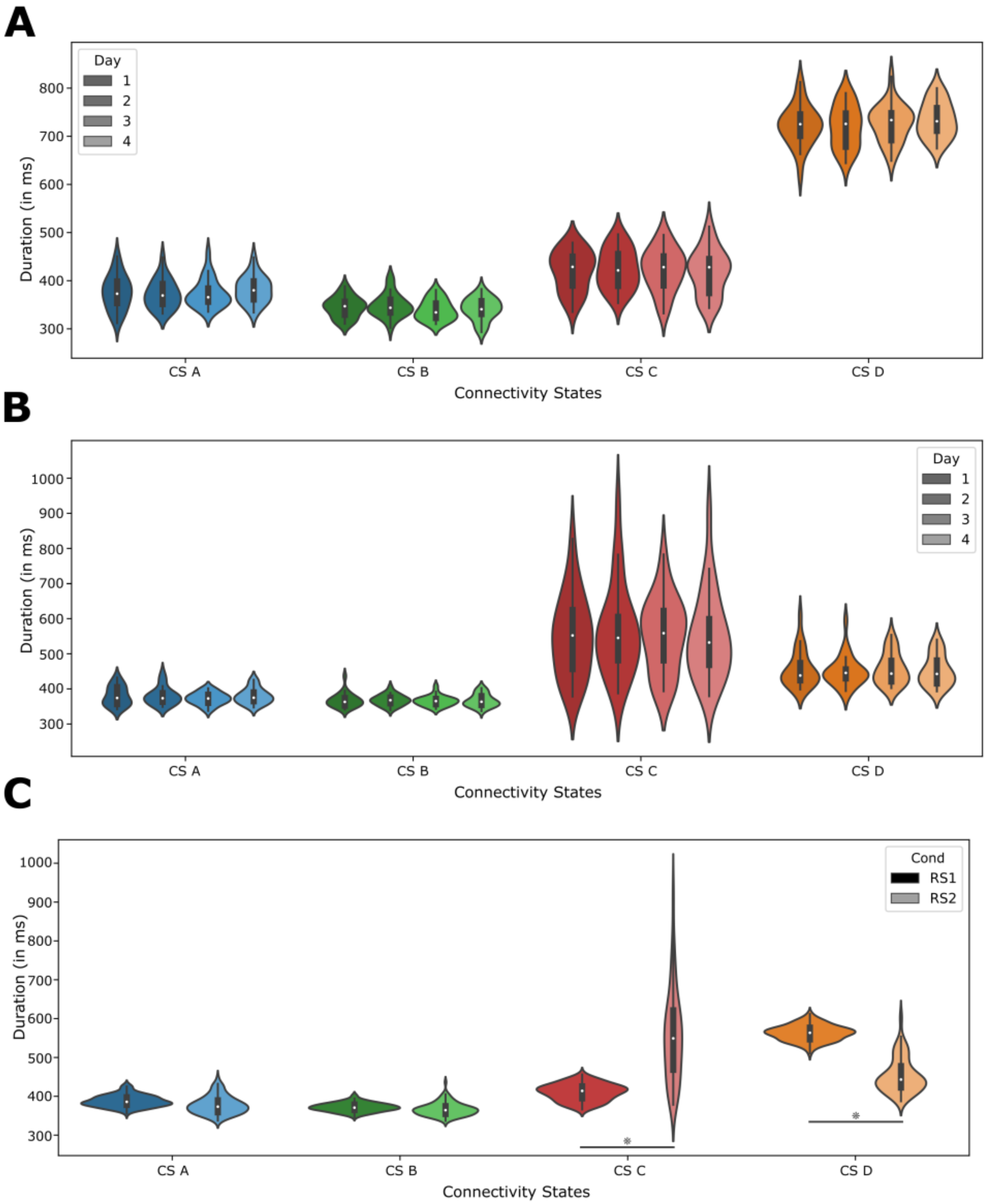

Fig S7. Connectivity state duration distribution. A: Connectivity state duration for states A-D over multiple measurement days in RS1; B: Connectivity state duration for states A-D over multiple measurement days in RS2; C: Comparison of connectivity state durations in RS1 and RS2 for states A-D.
